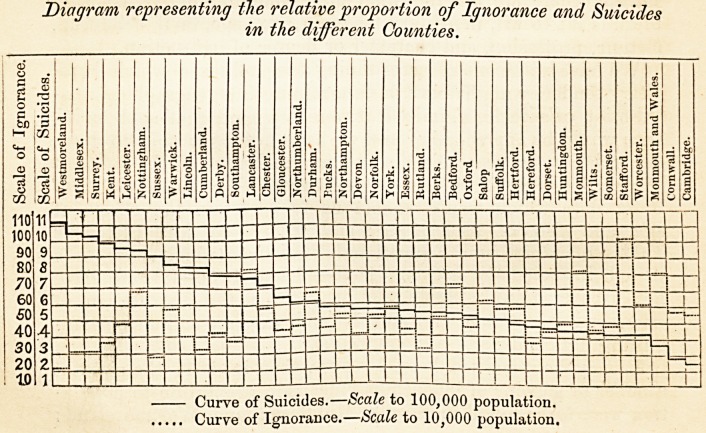# On the Distribution of Suicides in England and Wales

**Published:** 1859-10-01

**Authors:** 


THE JOURNAL

OF

PSYCHOLOGICAL MEDICINE

AND

MENTAL PATHOLOGY.

OCTOBER 1, 1859.

### Art. I.—ON THE DISTRIBUTION OF SUICIDES IN ENGLAND AND WALES.

(*With a Map.*)

When Virgil consigned the souls of suicides to a special region of Hades, and Dante planted them in a particular division of Hell, each poet represented as being a law of the infernal, what is in no small degree a law of terrestrial regions. There is, unhappily, no segment of England that is not blurred by suicides; but the number of suicides predominates conspicuously in certain districts as compared with others, so that we may map out particular localities in which there appears to be an extraordinary tendency to the act of self-destruction, and which may perhaps not unfittingly be designated *suicide-fields*.

The data at our disposal from which we may ascertain the distribution of suicides in the kingdom, are derived from the tabulated returns of inquests "touching the cause of death" contained in Mr. Sl. Redgrave's "Judicial Statistics," (*Blue Book*). These returns, made by the coroners throughout England and Wales, extend over a period of three years. In making use, however, of Mr. Redgrave's tabulated arrangement, in so far as it refers to counties, it is requisite to note that the boundaries of a county do not always represent the actual limits of a coroner's district; for it sometimes happens that the coroners of one county will, in cases of necessity, extend their jurisdiction into neighbouring counties, but the inquests so held are entered in the returns of the coroner who presides. There is no means of determining whether the errors arising from this irregularity affect materially or not the special results of the returns, but it is probable (as will appear subsequently) that they exercise but slight influence over the comparative results.

According to the coroners' returns, the following were the numbers of suicides which were committed in England and Wales during the years 1856–57–58 :—

| 1856. | | 1857. | | 1858. | |
|---|---|---|---|---|---|
| M. | F. | M. | F. | M. | F. |
| 919 | 395 | 960 | 389 | 909 | 366 |

These figures give an average of 6·8 suicides of both sexes in every 100,000 of the population of England and Wales during the three years 1856-58. The average of the five years, 1852-56, was, according to returns made to the Registrar-General, 5·8. In accounting for the difference between the two averages (putting aside the fact that the periods do not correspond), Dr. Farr's statements have to be considered, that the suicides returned to the Registrar-General are probably one-tenth below the actual number ascertained to have occurred, (an opinion doubtless founded upon a comparison with the coroners' returns for 1856), and that a few should be deducted from Mr. Redgrave's tables for "the duplicate return," (19*th Annual Report of the Registrar-General, p.* 203).

By a reference to the accompanying *Table* (I.), it will be seen that the proportion of suicides in fifteen counties is above the average of the kingdom (6·8); in one it is about the average; while in the remaining counties it is, in different degrees, below. The counties in which the proportion is in excess of the average are as follows :—

| | | | |
|---|---|---|---|
| Westmoreland...... | 11·1 | Warwick ......... | 8.5 |
| Middlesex ......... | 10·5 | Lincoln ......... | 8·4 |
| Surrey... ............ | 10·4 | Cumberland ... | 8·4 |
| Kent ............... | 9·9 | Derby ............ | 7·7 |
| Leicester..... ...... | 9·6 | Southampton ... | 7·7 |
| Nottingham ...... | 9·5 | Lancaster ...... | 7·6 |
| Sussex ............. | 9·0 | Chester ......... | 7·1 |
| Brecon ......... | 7·0 | | |

The average of Gloucestershire (6·5) is about that of the kingdom, and next in order of proportion to that county stand Northumberland (6·1) and Durham (6·1). The position of those counties, the averages of which range below 6·0, will be most readily seen by a reference to the Map which accompanies this article, and to *Table* I. Lowest in the scale are the Welsh counties of Carmarthen (1·7) and Pembroke (1·0).

I. *Table showing the Distribution of Suicides in the different Counties of England and Wales during the Three Years* 1856–57–58.

| — | 1856. | | 1857. | | 1858. | | Average of the Three Years. | Estimated Average Population. | Average of Suicides to 100,000 Population. | Proportion of Persons to 10,000 Population who signed the Marriage Register with Marks, 1857. |
|---|---|---|---|---|---|---|---|---|---|---|
| | Male. | Female. | Male. | Female. | Male. | Female. | | | | |
| Bedford . . . . | 4 | 7 | 2 | 4 | 2 | 2 | 7·0 | 134,402 | 5·3 | 73·2 |
| Berks . . . . | 6 | 8 | 1 | 2 | 9 | 3 | 9·2 | 175,045 | 5·4 | 50·3 |
| Buckingham . . | 11 | 5 | 3 | 2 | 8 | 1 | 10·0 | 168,091 | 5·9 | 45·4 |
| Cambridge . . . | 5 | 4 | 2 | 1 | 1 | 2 | 5·0 | 197,969 | 2·5 | 56·6 |
| Chester . . . . | 21 | 39 | 3 | 15 | 21 | 9 | 36·0 | 492,085 | 7·1 | 59·5 |
| Cornwall . . . | 8 | 5 | 2 | 3 | 7 | 5 | 10·0 | 363,592 | 2·7 | 58·6 |
| Cumberland . . | 14 | 14 | 4 | 3 | 13 | 4 | 17·3 | 205,962 | 8·4 | 33·6 |
| Derby . . . . | 14 | 24 | 8 | 8 | 13 | 5 | 24·0 | 310,412 | 7·7 | 43·0 |
| Devon . . . . | 31 | 10 | 11 | 13 | 28 | 10 | 34·3 | 587,576 | 5·8 | 40·3 |
| Dorset . . . . | 8 | 2 | 2 | 5 | 5 | 4 | 8·6 | 189,697 | 4·5 | 44·9 |
| Durham . . . | 16 | 24 | 9 | 7 | 15 | 10 | 27·0 | 440,815 | 6·1 | 67·3 |
| Essex . . . . | 12 | 16 | 5 | 2 | 19 | 11 | 21·6 | 383,916 | 5·6 | 43·0 |
| Gloucester . . . | 23 | 21 | 6 | 13 | 23 | 7 | 31·0 | 475,191 | 6·5 | 45·5 |
| Hereford . . . | 5 | 7 | 3 | 1 | 0 | 1 | 5·6 | 116,755 | 4·7 | 36·4 |
| Hertford . . . | 4 | 11 | 4 | 2 | 4 | 1 | 8·6 | 173,676 | 4·9 | 56·2 |
| Huntingdon . . | 1 | 4 | 1 | 3 | 0 | 0 | 3·0 | 67,561 | 4·4 | 48·8 |
| Kent . . . . . | 51 | 41 | 19 | 16 | 52 | 16 | 65·0 | 655,612 | 9·9 | 36·3 |
| Lancaster . . . | 136 | 129 | 54 | 46 | 114 | 36 | 171·6 | 2,249,744 | 7·6 | 82·2 |
| Leicester . . . | 14 | 20 | 6 | 3 | 20 | 6 | 23·0 | 238,972 | 9·6 | 47·3 |
| Lincoln . . . . | 23 | 24 | 15 | 13 | 24 | 10 | 36·1 | 427,994 | 8·4 | 40·6 |
| Middlesex . . . | 144 | 159 | 69 | 89 | 140 | 58 | 219·6 | 2,072,540 | 10·5 | 30·5 |
| Monmouth. . . | 5 | 8 | 1 | 0 | 5 | 4 | 7·6 | 171,248 | 4·4 | † |
| Norfolk . . . . | 16 | 30 | 14 | 9 | 8 | 4 | 27·0 | 460,744 | 5·8 | 53·9 |
| Northampton . . | 12 | 8 | 3 | 5 | 9 | 2 | 13·0 | 220,270 | 5·9 | 54·9 |
| Northumberland | 10 | 14 | 9 | 8 | 11 | 8 | 20·0 | 326,092 | 6·1 | 46·9 |
| Nottingham . . | 16 | 18 | 5 | 9 | 26 | 7 | 27·0 | 282,733 | 9·5 | 67·4 |
| Oxford . . . . | 5 | 7 | 2 | 2 | 7 | 4 | 9·0 | 174,825 | 5·1 | 45·2 |
| Rutland . . . . | 1 | 1 | 2 | 0 | 0 | 0 | 1·3 | 23,991 | 5·4 | 31·2 |
| Salop . . . . . | 13 | 5 | 1 | 2 | 11 | 3 | 11·6 | 231,453 | 5·0 | 62·6 |
| Somerset . . . | 18 | 11 | 4 | 5 | 12 | 4 | 18·0 | 418,902 | 4·2 | 47·8 |
| Southampton . . | 26 | 34 | 6 | 6 | 22 | 7 | 33·6 | 435,778 | 7·7 | 37·8 |
| Stafford . . . . | 26 | 14 | 5 | 4 | 25 | 11 | 28·3 | 664,260 | 4·2 | 100·5 |
| Suffolk . . . . | 16 | 18 | 3 | 2 | 8 | 6 | 17·6 | 350,499 | 5·0 | 56·2 |
| Surrey . . . . | 61 | 49 | 38 | 21 | 44 | 19 | 77·3 | 742,506 | 10·4 | 30·6 |
| Sussex . . . . | 20 | 23 | 8 | 9 | 31 | 7 | 32·6 | 358,900 | 9·0 | 29·9 |
| Warwick . . . | 28 | 34 | 14 | 14 | 28 | 16 | 44·6 | 518,999 | 8·5 | 58·4 |
| Westmoreland . | 6 | 3 | 3 | 2 | 5 | 1 | 6·6 | 59,385 | 11·1 | 22·3 |
| Wilts . . . . | 8 | 6 | 4 | 3 | 9 | 3 | 11·0 | 252,991 | 4·3 | 44·4 |
| Worcester . . . | 10 | 12 | 3 | 1 | 7 | 5 | 12·6 | 294,002 | 4·2 | 62·6 |
| York . . . . . | 73 | 71 | 30 | 25 | 94 | 42 | 111·6 | 1,921,553 | 5·8 | 59·1 |
| Anglesey . . . | 0 | 1 | 0 | 1 | 1 | 3 | 2·0 | 61,185 | 3·2 | † Monmouth and Wales. 81·5 |
| Brecon . . . . | 3 | 5 | 1 | 0 | 4 | 1 | 4·6 | 64,996 | 7·0 | |
| Cardigan . . . | 1 | 1 | 4 | 1 | 1 | 0 | 2·6 | 72,014 | 3·6 | |
| Carmarthen . . | 3 | 2 | 0 | 1 | 0 | 0 | 2·0 | 113,212 | 1·7 | |
| Carnarvon . . . | 3 | 4 | 1 | 1 | 2 | 0 | 3·6 | 91,932 | 3·9 | |
| Denbigh . . . | 0 | 3 | 1 | 0 | 2 | 0 | 2·0 | 95,043 | 2·1 | |
| Flint . . . . . | 0 | 2 | 0 | 0 | 3 | 1 | 2·0 | 68,894 | 2·9 | |
| Glamorgan . . | 3 | 9 | 3 | 5 | 11 | 5 | 12·0 | 268,245 | 4·4 | |
| Merioneth . . . | 1 | 1 | 0 | 1 | 0 | 0 | 1·0 | 38,555 | 2·6 | |
| Montgomery . . | 3 | 2 | 0 | 0 | 2 | 1 | 2·6 | 65,973 | 3·9 | |
| Pembroke . . . | 1 | 0 | 0 | 1 | 1 | 0 | 1·0 | 97,794 | 1·0 | |
| Radnor . . . . | 0 | 0 | 0 | 0 | 2 | 1 | 1·0 | 24,272 | 4·1 | |
| England and Wales . . | 919 | 395 | 960 | 389 | 909 | 366 | 1312·6 | 19,108,508 | 6·8 | |

If the foregoing figures be regarded as a measure of tendency to suicide, it would appear that in the three years, 1856-58, the maximum disposition to self-destruction was manifested in Westmoreland; that next in rank, and hardly inferior to Westmoreland, stood the metropolitan counties of Middlesex and Surrey; while the minimum disposition was shown in Carmarthen and Pembroke.

From the general distribution of suicides throughout the kingdom, we learn that there are three districts in which the tendency to suicide is in excess. For the convenience of description we propose to designate these districts *suicide-fields*, and to distinguish the one from the other by their relation to the metropolis, the midland, and the northern counties.

I. *The London Suicide-field.*—The metropolitan counties of Middlesex, Surrey, and Kent, and the adjoining counties of Sussex and Hampshire, form a well-defined district in which the proportion of suicides is considerably in excess of that of the whole kingdom.

If (as in the case of the departments adjoining Paris) the excess of suicides in this district is to be ascribed mainly to the influence of the metropolis, it is remarkable that that influence should be manifested only in the counties south of the metropolis, being confined on the north, north-east, and north-west, within the limits of Middlesex, and on the west not extending beyond the boundaries of Surrey. Paris forms the *centre* of a vast suicide field, and M. Guerry has remarked, and his remark has been confirmed by subsequent writers, that, generally speaking, from whatever point of France we start, the number of suicides increases regularly in proportion as we approach the capital.—(*Essai sur la Statistique Morale de France.* p. 65. 1838.) M. Brierre de Boismont states that, "It appears certain that the moral action of the capital radiates from a central point towards the surrounding districts." And again, "It may be regarded as a fact perfectly established, that the number of suicides increases regularly and in all directions, in proportion as Paris is neared."—(*Du Suicide et de la Folie Suicide*, pp. 356-357.) Marseilles appears to affect Provence and Dauphinè much in the same manner as Paris affects the rest of France.

It can hardly be doubted that the excess of suicides in the metropolitan counties—Middlesex, Surrey, and Kent,—is due to the metropolis; and probably the peculiarity of the social relations of London to Sussex, and even Hampshire, may account for the excessive suicidal tendency manifested in those counties, as well as for the southerly extension of the baneful influence of the metropolis.

The sea-board of Sussex is one of the most favoured places of resort, and one much frequented, for its readiness of access, by Londoners; and it is a suggestive fact that of ninety-eight suicides which were committed in Sussex in the three years, 1856-58, *thirty-nine*, that is to say, considerably more than one-third (in fact, 40·8 per cent.) occurred within the boroughs of Brighton and Hastings, and the rape of Hastings,—Brighton numbering twenty suicides, and Hastings and its rape eighteen.

It may be questioned to what extent Hampshire is affected by the metropolis, since there are local causes in operation at Portsmouth and Southampton which might alone elevate the average of suicides in that county to its present pitch, and thus perhaps constitute Portsmouth and its vicinity a species of secondary suicide-field. Of 101 suicides committed in Hampshire in 1856–58, forty-two were committed in the boroughs of Portsmouth (24), Southampton (7), and in the Isle of Wight (11). Still the intercourse between the sea-board of Hampshire and London is so intimate and peculiar, that we must not lightly put aside the probability of suicidal infection from the metropolis, particularly as it would appear that while the suicides in the borough of Portsmouth form 24·0 per cent. of the suicides of Hampshire, those of the borough of Plymouth form for the same period but 13·5 per cent. of the suicides of Devonshire, and those of the borough of Liverpool 15·5 per cent. of the suicides of Lancashire.

The excessive tendency to suicide in the borough of Portsmouth as compared with the other great sea-port boroughs named, relatively to their counties, is sufficiently remarkable. The suicides in the borough of Kingston-upon-Hull form only 5·0 per cent. of those of Yorkshire, but we should perhaps have the proportion of suicides in this sea-port borough relatively to those of the East-Riding of Yorkshire, in order to make a legitimate comparison with Portsmouth.

Until 1858, we possessed no very trustworthy data of the actual number of suicides occurring in the metropolis itself. In and since that year, however, suicides have been distinguished in the Registrar-General's Weekly Bills of Mortality, and it would appear that, in the twelve months of 1858, 283 individuals committed self-murder in London, giving an average of 9·2 in every 100,000 of the population.

II. *The Midland Suicide-field.*—Leicestershire, Nottinghamshire, Warwickshire, and Lincolnshire constitute a well-marked suicide-field, the centre of which is formed by Notts (9·5), and Leicestershire (9·6), in which counties the tendency to suicide is greatest, and about equal—while the extremities are constituted by Lincolnshire (8·4), and Warwickshire (8·5), the tendency in these counties being also about equal, but less than in the two previously named counties. There is a considerable falling off of intensity in adjoining counties, the diminution, however, being less marked in Derbyshire (7·7), and in Gloucestershire (6·5), the tendency to suicide in the latter county being about the average of the whole kingdom (6·8).

III. *The Northern Suicide-field.*—This field is formed by

Westmoreland (11·1), Cumberland (8·4), and Lancashire (7·6). To meet the exigencies of a shaded scale, in the Map Northumberland, Durham, and Cheshire are darkened to the same degree as Lancashire; but a reference to *Table* I., or to the figures marked in the Map within the counties named, will at once show that they are rightly excluded from entering into the formation of the suicide-field. In the two first-mentioned counties, the tendency to suicide barely exceeds that of Yorkshire, and is below the average of the kingdom; in Cheshire it is about the average. It is to be regretted that the coroners' returns do not show the proportion of suicides in the different Ridings of Yorkshire, but, so far as can be ascertained from the said Returns, it would seem as if the average of suicides of the West Riding at least, did not differ much from the general average of the county.

The excessive proportion of suicides in Westmoreland, and the greater diminution of tendency to suicide in Lancashire, as compared with Cumberland, are interesting facts. It will, however, be matter for subsequent inquiry, how far the great apparent tendency to suicide in Westmoreland and Cumberland may be determined by causes not inherent in the counties. For instance, both counties, on account of the beauty of their scenery, are greatly resorted to from neighbouring counties, and, indeed, from the kingdom generally. Is the proportion of suicides exaggerated by suicides among casual visitors, or temporary residents?

*Should Breconshire be described as a Suicide-field?* Certainly the average of suicides occurring in it (7·0), is barely in excess of the average of the kingdom (6·8), but it is singularly in excess as compared with that of the Welsh counties (3·3); hence, if subsequent returns should show the persistence of this excess, and the continuance of the present isolation, on account of that excess, from neighbouring counties, Brecon may rightly be termed a suicide-field.

We should have felt some hesitation in describing the foregoing suicide-fields as such, and as if they were probably persistent features of the distribution of suicides in the kingdom, upon the authority of the coroners' returns alone. It would appear, however, from an analysis of the returns of suicides contained in the Registers of Deaths for 1838–9, made by Dr. Farr, and published in the Third Annual Report of the Registrar-General (p. 75, *et seq.*), that the districts of greatest excess of suicides in the two years mentioned were the same as in the three years 1856–8. This important and interesting fact affords strong presumptive evidence that the localities of greatest tendency to suicide are, comparatively speaking, persistent.

The following Table shows the average proportion of suicides in the different registration districts in the two periods 1838–9

and 1856–8. It is necessary to remark, however, that this comparison is approximative only in the London and South-Eastern districts. London not being specified apart from the Metropolitan Counties' Returns in the coroners' lists, it has been necessary to unite Surrey and Middlesex together to form the London district, and Kent, Sussex, and Hampshire to form the South-Eastern district. The great preponderance of suicides in these districts, however, in both series of averages, shows that the want of accurate correspondence in the character of the divisions does not affect the comparative result. Probably also, a want of narrow agreement between the boundaries of the registration districts and counties, and the ordinary boundaries of counties as summed up according to registration districts, will not affect materially the general results obtained, from a comparison of the averages derived from the Registrar-General's and the Coroners' Returns.

II. *Table showing the Distribution of Suicides in the different Registration Divisions, in the two Periods,* 1838–39, *and* 1856–58.

| Registration Divisions. | I. London. | II. South-Eastern Counties. | III. South-Midland Counties. | IV. Eastern Counties. | V. South-Western Counties. | VI. West-Midland Counties. | VII. North-Midland Counties. | VIII. North-Western Counties. | IX. Yorkshire. | X. Northern Counties. | XI. Monmouth and Wales. | England and Wales. |
|---|---|---|---|---|---|---|---|---|---|---|---|---|
| Average Annual Cases of Suicides to 100,000 Inhabitants, 1838—39. | 10·9 | 8·4 | 5·8 | 5·7 | 5·3 | 4·4 | 6·8 | 6·5 | 5·2 | 6·5 | 2·2 | 6·3 |
| Average Annual Cases of Suicides to 100,000 Inhabitants, 1856—58. | 10·5 | 8·6 | 4·8 | 5·5 | 4·4 | 5·8 | 8·6 | 7·5 | 5·8 | 6·5 | 3·6 | 6·8 |

If the figures for the two periods contained in the foregoing Table be compared, it will be seen that the averages of the Metropolitan and South-Eastern districts scarcely vary; that the North Midland district holds the same relation to the other districts in both periods, although the average of the last period has increased one-third; that the Northern and North-Western districts have also the same comparative relationship in the two series of averages, although the average of the North-Western district is higher in 1856–9 than in 1838–9, that of the Northern district being the same in both periods. It is seen also that the averages of the South-Midland and South-Western districts have diminished, while the averages of the Eastern and remaining districts vary but slightly, except Monmouth and South Wales, the average of which district, notwithstanding that it still remains the lowest in the kingdom, has increased one-third.

Thus far, we have dealt with the results derived from the total number of suicides of both sexes. If, however, we separate the one sex from the other, several interesting particulars will be ascertained.

The average proportion of suicides in every 100,000 of the *male* population during 1856–9 was 9·9; of the *female*, 3·9. Now if a reference be made to the following *Table* (III.), it will be seen that the proportion of *male* suicides was above the average of the kingdom in two districts only, the Metropolitan and South-Eastern, the proportion being above the average in the North Midland. But the proportion of *female* suicides was above the average in no less than *eight* of the *eleven* districts, to wit, the Metropolitan, South-Eastern, Eastern, West-Midland, North-Midland, North-Western, Yorkshire, and Northern, while it was at the average in one, the South-Midland. Moreover, the proportion of male and female suicides was about equal in three divisions, the South-Midland, Eastern, and Monmouth and Wales; the proportion of males was but slightly in excess of females in the Northern district; and it varied only from one-fourth to one-third in excess in the remaining districts.

III. *Table showing the Distribution of Suicides of each Sex in the different Registration Counties*, 1856–58.

| | Registration Counties. | Average to 100,000 population of each sex. | |
|---|---|---|---|
| | | Males. | Females. |
| I. | London | 12·5 | 8·1 |
| II. | South-Eastern Counties | 10·3 | 6·6 |
| III. | South-Midland Counties | 4·7 | 3·9 |
| IV. | Eastern Counties | 5·8 | 5·5 |
| V. | South-Western Counties | 5·9 | 3·0 |
| VI. | West-Midland Counties | 6 8 | 4·4 |
| VII. | North-Midland Counties | 9·8 | 7·7 |
| VIII. | North-Western Counties | 8·7 | 6·4 |
| IX. | Yorkshire | 6·9 | 4·8 |
| X. | Northern Counties | 7·2 | 6·1 |
| XI. | Monmouth and Wales | 3·5 | 3 3 |
| | ENGLAND AND WALES | 9·9 | 3·9 |

In addition to the facts already set forth from the coroner's returns, there is another well worthy of being noted, namely, the great fluctuations in the number of suicides from year to year in several counties. Thus, for example, 60 suicides were committed in Cheshire in 1856, 18 in 1857, and 30 in 1858; 60 were committed in Hampshire in 1856, 12 in 1857, and 29 in 1858. Other illustrations may be obtained by referring to *Table* I. If the coroners in one or more of the counties where these great variations are observed, could be induced to record carefully the circumstances attending and determining the act of self-destruction, doubtless much light would be thrown upon the influences which govern the prevalence of suicide.

If we seek now to ascertain the causes which lead to the peculiarities of distribution of suicides in England and Wales, we are at the very outset confronted with difficulties which are for the present insuperable. The data which we have made use of in this article enable us to determine the general facts of the greater or less prevalence of suicides in different localities; but they furnish no particulars by which we might eliminate errors, or with any information concerning the age, the civil condition of the suicides, or the circumstances which prompted the act of self-destruction. We can only then endeavour to ascertain if the general knowledge we possess of the etiology of suicide will in any way aid us in elucidating this subject.

Brierre de Boismont (*Op. cit.*, p. 100) collated the causes which led to the committal of suicide in 4077 cases, and with the following results:—

| | | | |
|---|---|---|---|
| Insanity | 15·9 per cent. | Hypochondriasis | 3·2 per cent. |
| Drunkenness | 12·9 „ | Remorse, fear of dishonour or of justice | 2·9 „ |
| Diseases | 9·9 „ | Misconduct | 1·2 „ |
| Domestic troubles | 8·8 „ | Idleness | 1·3 „ |
| Chagrin, disappointment | 7·6 „ | Acute delirium | 1·3 „ |
| Love | 7·4 „ | Gaming | 1·0 „ |
| Poverty, misery | 6·9 „ | Want of work | 1·0 „ |
| Pecuniary difficulties | 6·8 „ | Pride and vanity | 0·67 „ |
| Ennui | 5·8 „ | Divers motives | 0·97 „ |
| Feebleness, exaltation, sadness | 3·5 „ | | |

Of any single, well-defined cause of suicide, insanity, according to the foregoing list, plays the foremost part. Does insanity manifestly contribute in determining the position of the districts of greatest excess of suicides in England? The only statistics accessible by which an approximative knowledge of the distribution of unsoundness of mind throughout the kingdom may be obtained, are those contained in the returns of pauper lunatics chargeable to parishes, furnished at intervals by the Poor Law Board to the Commissioners in Lunacy. The last return made of this character is for the year 1857,* and it is contained in

* The 13th Report of the Commissioners in Lunacy, recently published, contains an abstract of the "Annual Returns of Pauper Lunatics and Idiots belonging to the several Unions in *England* and *Wales* on the 1st of January, 1859."

the Twelfth Annual Report of the Lunacy Commissioners. From this return it would appear that the greatest proportion of pauper lunatics is found in Gloucestershire and Berkshire; next in the scale stand the counties of Middlesex, Surrey, Hertfordshire, Buckinghamshire, Oxfordshire, Wiltshire, Northamptonshire, and Shropshire; while in reference to the counties of less tendency (confining our attention to the suicide-fields), Kent and Hampshire hold a third place in the list (this being divided into five classes), and Sussex a fourth, in the London field; Nottingham and Warwick also hold a third place, and Lincolnshire a fourth, in the Midland field; and Cumberland a third, and Westmoreland and Lancashire a fourth in the Northern field. Thus it will be seen that there is no systematic agreement between the counties of greatest tendency to lunacy and the counties of greatest tendency to suicide. Of the counties standing prominent in the suicide-fields, Middlesex, Surrey, and Leicester alone appear as manifesting a more than average tendency to lunacy.

With the exception of drunkenness, no one of the remaining determining causes of suicide is so prominent that it might be expected to exert a marked influence upon the geographical distribution of the act, and the promptings of drunkenness are so often interwoven with the moral causes which too commonly bring about intemperance, that it cannot very well be separated from the moral causes of suicide.

Suicide is, indeed, an exceptional result of the disease and wretchedness, the many bitter pangs and troubles which infest life, and a tabulated list of the causes which have apparently immediately brought about the act of self-destruction, presents but a reflex of every-day events and evils. Are we then to look upon suicide as an index of the degree of tension of those moral and physical causes which predispose to and determine it? This proposition may involve a truism, but if we assume it, we shall find that it will aid us but little in our quest, and that we fall short of some element or elements in the causation of self-murder.

It is certain that the greatest tendency to suicide is found for the most part in districts which are chief centres of commercial activity, and where the mental, moral, and physical powers are kept in the highest degree of tension. This is true of London; of Leicestershire, Nottinghamshire, and Warwickshire; of Cumberland and Lancashire; and the seemingly anomalous positions of Westmoreland, Lincolnshire, Kent, Sussex, and Hampshire may perhaps be subsequently explained by suicidal infection from the counties first named. But, admitting this, Gloucestershire, Derbyshire, Staffordshire, and Shropshire, Cheshire, Durham, and Northumberland, and we believe also the West Riding of Yorkshire, all localities second to none of the suicide-fields, except perhaps the London field, in their commercial activity, and in the turmoil and tension of life in them, exhibit but a slight tendency towards self-destruction.

Differences in the character of the industry of different counties do not apparently solve the question. The tendency to suicide is below the average in the purely agricultural counties, with the exception of Lincoln, Kent, Sussex, and Hampshire. In Herefordshire, Buckinghamshire, Bedfordshire, and Somerset, counties in which agricultural pursuits predominate, but in which small manufactures are carried on in cottages, such as lace-making, straw-plaiting, and glove-making (Somersetshire), and in which immorality and ignorance are great, suicide is also below the average. If we compare the agricultural and mining counties of Westmoreland, Cumberland, Northumberland, and Durham, with the agricultural and mining counties of Cornwall, Monmouth, and the districts of North and South Wales, we see the northern counties ranging towards the highest pitch of suicidal disposition, the southern and Welsh towards the bottom of the scale. Of the great manufacturing and mining counties of Lancashire, Cheshire, Staffordshire, Derbyshire, Nottinghamshire, Leicestershire, Warwickshire, Worcestershire, Gloucestershire, and the great manufacturing district of the West Riding of Yorkshire, we find Gloucestershire, Staffordshire, and Worcestershire, and probably also the West Riding of Yorkshire, manifesting a much inferior disposition to suicide than the other counties.

But, in addition to the causes which determine the act of suicide, there are others which are influential in predisposing towards it; to wit, hereditary tendency, sex, age, civil condition, fortune, profession, and character or degree of instruction.

We have no means of ascertaining the influence which hereditary tendency might have in determining the geographical distribution of suicide, if, indeed, it were supposed that such influence would be appreciable; and we have not been able to trace any relation between the distribution and that of the different sexes, of celibates, widows, or widowers. We entirely fail of those data which would enable us to ascertain the effects of age, and also of professions; and, guided by Mr. Fletcher's Tables of Moral and Educational Statistics (which refer to a period intermediate between 1838–39 and 1856–58), we have not discovered any intimate relation between the distribution of crime and immorality and suicide (and, we may add also, as requiring notice, density of population).

It may be remarked here, however, that in 1841 the greatest number of persons of independent means in proportion to population were to be found in Surrey, Middlesex, and Westmoreland; and in 1842–43, the greatest amount of real property existed in Lincolnshire—all counties of excessive suicidal tendency. These coincidences deserve to be remarked in connexion with the opinion of some writers, that wealth and a sufficiency of means predispose to suicide—in fact, that a greater tendency to suicide is manifested among those of easy circumstances than among the impoverished. Thus Dr. Marc d'Espine remarks on the suicide-statistics of the Canton of Geneva:—" If we consider the suicides apart, we find that thirteen were wealthy individuals, thus giving 1·85 per cent. as the lethiferous figure of the wealthy, in place of 1·21 per cent. the lethiferous figure of the whole population. Another calculation leads to the same result. The deaths among the rich form 4·2 per cent. of the whole mortality, and suicides among the rich form 6·3 per cent. of the total deaths from suicide. Easy circumstances then increase the chances and occasions of suicide."—(*Statistique Mortuaire Comparée*, p. 114.)

In one respect only do we find any general correspondence between a predisposing cause of suicide and its geographical distribution. It is a fact of singular interest that suicide prevails most in the most educated districts. If we adopt as a measure of the degree of ignorance of different counties, the number of persons who sign the marriage register with marks, we find, as a general rule, that the *average number of suicides decreases as the average amount of ignorance increases.*

The relationship existing between suicide and ignorance will be best seen by a reference to the accompanying diagram, in which is depicted the proportion of suicides and the degree of

*Diagram representing the relative proportion of Ignorance and Suicides in the different Counties.*

—— Curve of Suicides.—*Scale* to 100,000 population.
..... Curve of Ignorance.—*Scale* to 10,000 population.

ignorance in the several counties of England, and in the registration division of Monmouth and South Wales, as set forth in *Table* I. The lines of suicide and ignorance describe curves in opposite directions, and although the points of depression of the one curve and of elevation of the other do not absolutely agree, still the correspondence is such as, perhaps, to justify the conclusion that *the prevalence of suicide and the degree of ignorance in a district are in inverse relation the one to the other.*

This, which is true of 1856-58, is also true in the main of 1838-39. Dr. Farr writes :—" Suicide is, in fact, most frequent in the metropolis, the south-eastern counties, and the northern counties, where the greatest number can write, and it is the least frequent in Wales. The intermediate counties range from 62 to 48, who could write, in 100 (persons married), the suicides from 4·5 to 6·8 in 100,000." The proportion who could write, in the metropolis, was 82 per cent., in the northern counties, 68, in the south-eastern, 62, and in Monmouth and Wales, 41.—(*Third Report of the Registrar-General*, p. 80.)

The general relationship which exists between the amount of instruction in the different French departments, and the number of suicides occurring in them, is similar to that found in the English counties. But, in addition to this, the French statistics of suicide and instruction for the thirteen years 1836-48 show, that *suicide has progressively increased in frequency in proportion as instruction has become more diffused in each region, as well as throughout the whole of France.* M. Lisle, from whose valuable work, *Du Suicide, Statistique, Médecine, Histoire, et Legislation* (Paris, 1856, p. 79), the foregoing conclusions are derived, asserts also, that wherever a comparative examination of the relation of suicide to instruction has been made, the results have proved absolutely similar to those obtained in France. He quotes the following Table from a memoir, by M. Brouc (*Annales d'hygiène publique et de médecine légale*, t. xvi.)

*Table showing the Relation of Suicide to the State of Instruction in different Countries.*

| Names of Cities or Countries. | Proportion of Scholars to Inhabitants. | Proportion of Suicides to Inhabitants. | Average of Scholars. | Average of Suicides. |
|---|---|---|---|---|
| Boston . . . | 1 in 3·5 | 1 in 12·500 | 1 in 5·6 | 1 in 12·644 |
| New York . . | 1 ,, 3·9 | 1 ,, 7·797 | | |
| Prussia . . . | 1 ,, 7·0 | 1 ,, 14·404 | | |
| Philadelphia . | 1 ,, 8·0 | 1 ,, 15·875 | | |
| Austria . . . | 1 ,, 13·0 | 1 ,, 20·900 | 1 in 132 | 1 in 30·274 |
| France . . . | 1 ,, 17·0 | 1 ,, 20·740 | | |
| Russia . . . | 1 ,, 367·0 | 1 ,, 49·182 | | |

The figures in this Table are derived from M. Balbi's work, *La Monarchie Française comparée aux principaux Etats du Globe,* and they refer to the interval comprised between the years 1827 and 1834, but it is not improbable that later inquiries would have furnished similar results, and M. Lisle thinks that he is warranted in assuming from his own and M. Brouc's researches, that for a *considerable period the frequency of suicides has been in the direct ratio of the state of instruction.**

Of 3086 cases of suicide in which the degree of instruction was ascertained by Brierre de Boismont (*Op. cit.* p. 85), it was found that the education of 18·5 per cent. was good; that 25·5 per cent. read and wrote well; that 53·7 per cent. read and wrote without orthography, or read without writing; and that 2·1 per cent. were entirely without instruction.

Suicides and criminals have this in common, that a very large proportion of both classes are very imperfectly educated; and the conclusion to be derived from this fact is doubtless the same in both instances, namely, that much of the instruction in vogue is accompanied by moral influences of a very doubtful character, or is indeed entirely deficient in the elements of a sound moral training.

The preponderance of instruction among suicides, and the preponderance of suicides in instructed districts, are facts which probably explain each other, and they lead to the deduction that the differential element in the etiology of suicide,—that element which determines the act of destruction as an exceptional result of the wide-spread causes to which it is ordinarily attributed—is to be sought in a peculiar vicious or morbid tone of thought. Hence on this view the number of suicides occurring in a locality would be an index of the degree of prevalence of this mental perversion. The character of the perversion, assuming that it exists, and the circumstances which foster it, must be a subject of specific investigation.

The imperfection of our data interposes insurmountable obstacles in the way of any satisfactory inquiry into the causes which determine the different degrees of prevalence of suicide in different districts, and we are constrained, however unwillingly, to confine

* There is a general but no constant relation between the state of education thus tested, and the commission of suicide. It may be admitted that there is some relation between the development of the intellect and self-destruction; but the connexion must be in a great measure indirect and accidental. In opposition to the arguments derived from agricultural districts, and labourers in towns, there is the fact that suicide is more frequent among several classes of artisans, than it is among better educated people. If the progress of civilization is to be charged with the increase of suicide, we must therefore understand by it the increase of tailors, shoemakers, the small trades, the mechanical occupations, and the incidental evils to which they are exposed, rather than the advancement of truth, science, literature, and the fine arts.—*Dr. Farr. Op. Cit.* p. 80.

ourselves to an indication of the general and probable bearing of the scanty particulars which we possess capable of throwing any light upon the subject. The question is a complex one, and it is not unlikely that it may be found that the excessive tendency to suicide in certain localities is dependent not upon one cause only, but upon a combination of causes which may not be the same in each locality.

For the present, the facts we have detailed showing the distribution of suicides in England and Wales, must be regarded simply as facts, the full significance of which has still to be ascertained.

---

## Figures and Tables

**Figure f1:**